# Response to Fortin-Bédard et al. “User expectations and experiences of an assistive robotic arm in amyotrophic lateral sclerosis: a multicenter observational study”

**DOI:** 10.1186/s42466-025-00405-z

**Published:** 2025-09-26

**Authors:** Susanne Spittel, Thomas Meyer, Ute Weyen, Torsten Grehl, Patrick Weydt, Robert Steinbach, Susanne Petri, Petra Baum, Moritz Metelmann, Anne-Dorte Sperfeld, Dagmar Kettemann, Jenny Norden, Annekathrin Rödiger, Benjamin Ilse, Julian Grosskreutz, Barbara Hildebrandt, Bertram Walter, Christoph Münch, André Maier

**Affiliations:** 1 Charité– Universitätsmedizin Berlin, Department of Neurology, Center for ALS and other Motor Neuron Disorders, Berlin, Germany; 2 Ambulanzpartner Soziotechnologie APST GmbH, Berlin, Germany; 3 Berufsgenossenschaftliches Universitätsklinikum Bergmannsheil, Department of Neurology, Center for ALS and other Motor Neuron Disorders, Bochum, Germany; 4 Alfried Krupp Krankenhaus, Department of Neurology, Center for ALS and other Motor Neuron Disorders, Essen, Germany; 5 Department for Neuromusclar Disorders, Bonn University, Bonn, Germany; 6 Department of Neurology, Jena University Hospital, Jena, Germany; 7 Department of Neurology, Hannover Medical School, Hannover, Germany; 8 Department of Neurology, Universitätsklinikum Leipzig, Leipzig, Germany; 9 Department of Neurology, Sächsisches Krankenhaus Altscherbitz, Altscherbitz, Germany; 10 Department of Neurology, Universitätsmedizin Schleswig-Holstein, Campus Lübeck, Lübeck, Germany

Dear Editor,

We would like to express our appreciation to Fortin-Bédard et al. for their interest in our recently published article *“User expectations and experiences of an assistive robotic arm in amyotrophic lateral sclerosis: a multicenter observational study”* (Spittel et al., 2024). In particular, we thank Fortin-Bédard et al. for their thoughtful and constructive response to our published article and for the valuable contributions their group has made to the field.

We strongly acknowledge that the wider literature on the use of assistive robotic arms in people with severe upper limb disabilities beyond the ALS population has been highlighted by Fortin-Bédard et al. The detailed overview of the extensive scientific literature on the use of assistive robotic arms in populations with severe upper-limb disabilities outside of the ALS context significantly enriches the discourse on this topic. By highlighting studies involving individuals with muscular dystrophy, spinal muscular atrophy, cerebral palsy, and spinal cord injury, Fortin-Bédard et al. offer a valuable perspective that widens the understanding of the potential and impact of such technologies across all relevant indication, like ALS.

However, our original study was intentionally focused on the ALS population, as data regarding specific user expectations and experiences in this group remain limited. With the effort made by Fortin-Bédard et al. to contextualize their findings within a larger body of evidence, it is reasonable to assume an improvement in the quality of life of those affected by ALS. The comprehensive synthesis provided in the letter underscores shared outcomes such as enhanced participation in daily activities, increased user satisfaction, and reduction in caregiver burden—outcomes that are relevant and transferable to the ALS population.

This contribution not only reinforces the findings of the original study but also supports the external validity of assistive robotic arm applications more broadly. While we are grateful for this important addition to advancing research and dialog in this evolving and impactful area, we encourage further research needs to promote the widespread use of this important assistive device.

Sincerely,

Susanne Spittel

(on behalf of the authors)

